# Supplementary material for: Investigation of the Effect of Double-Filler Atoms on the Thermoelectric Properties of Ce-YbCo4Sb12
Source: Materials (Basel). 2023 May 18;16(10):3819. doi: 10.3390/ma16103819 (PMC10222194; doi:10.3390/ma16103819)
Supplement: Supplementary file 1 [file materials-16-03819-s001.zip › materials-2350466-supplementary.pdf]

Supplementary data

Table S1: The theoretical and actual element composition of $Ce_xYb_{0.2-x}Co_4Sb_{12}$

**Nominal**	**Actual**
$Yb_{0.2}Co_4Sb_{12}$	$Yb_{0.21}Co_{3.77}Sb_{12}$
$Ce_{0.025}Yb_{0.175}Co_4Sb_{12}$	$Ce_{0.01}Yb_{0.18}Co_{3.81}Sb_{12}$
$Ce_{0.05}Yb_{0.15}Co_4Sb_{12}$	$Ce_{0.04}Yb_{0.13}Co_{3.8}Sb_{12}$
$Ce_{0.075}Yb_{0.125}Co_4Sb_{12}$	$Ce_{0.05}Yb_{0.05}Co_{3.78}Sb_{12}$
$Ce_{0.1}Yb_{0.1}Co_4Sb_{12}$	$Ce_{0.13}Yb_{0.05}Co_{3.88}Sb_{12}$

EDS data

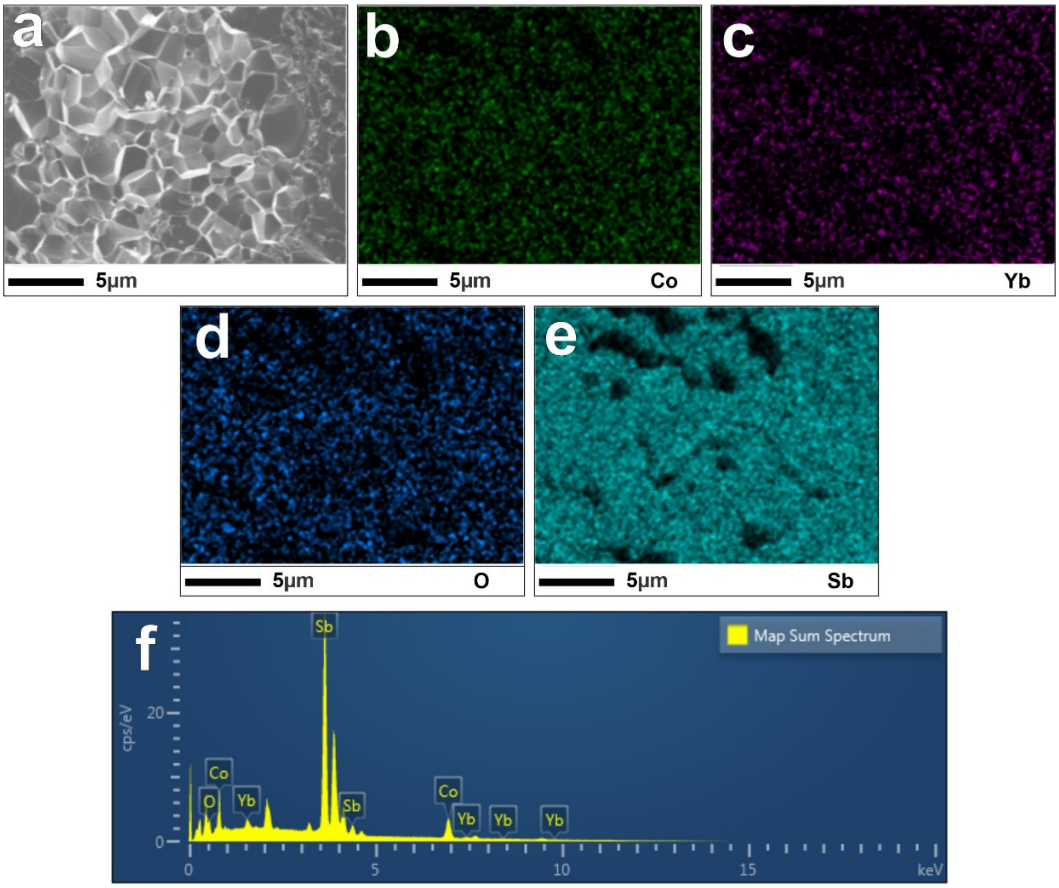

Figure S1: The (a) the SEM image, the distribution of (b) Co, (c) Yb, (d) O, (e) Sb, and (f) EDS map spectrum data of sample $Yb_{0.2}Co_4Sb_{12}$

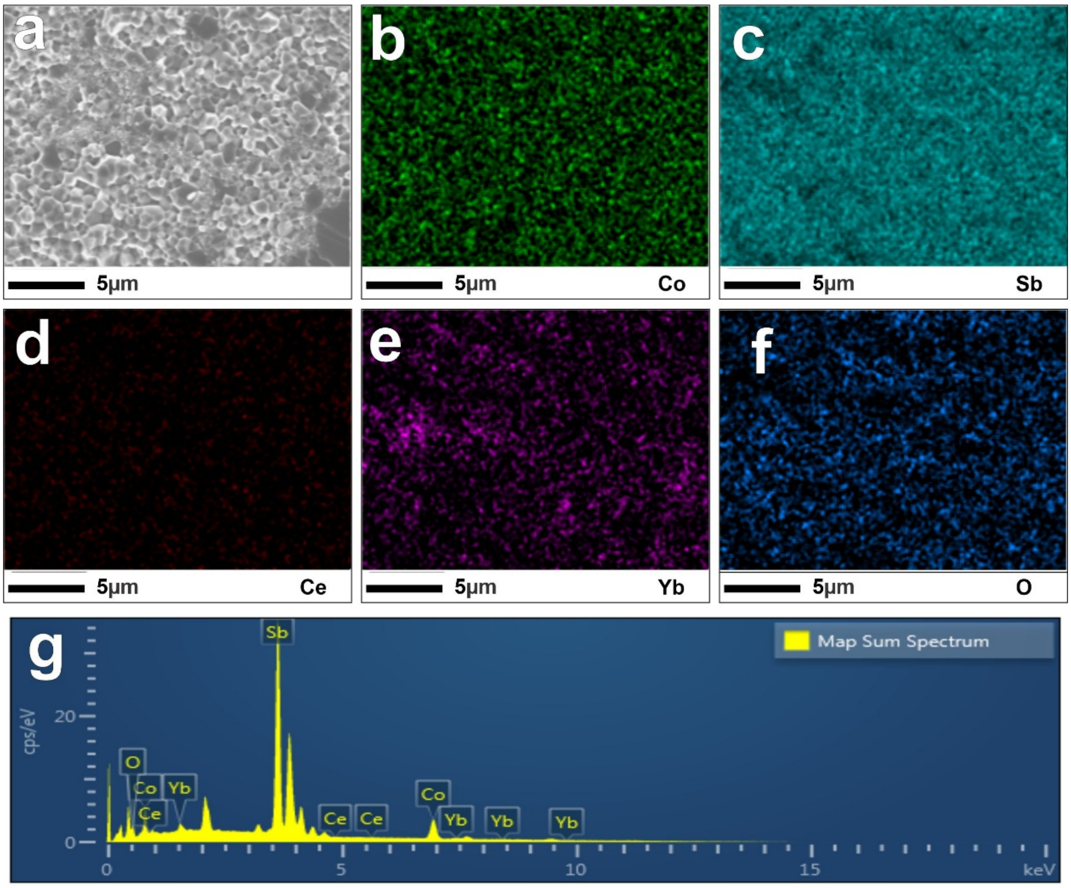

Figure S2: The (a) the SEM image, the distribution of (b) Co, (c) Sb, (d) Ce, (e) Yb, (f) O, and (g) EDS map spectrum data of sample $Ce_{0.025}Yb_{0.175}Co_4Sb_{12}$

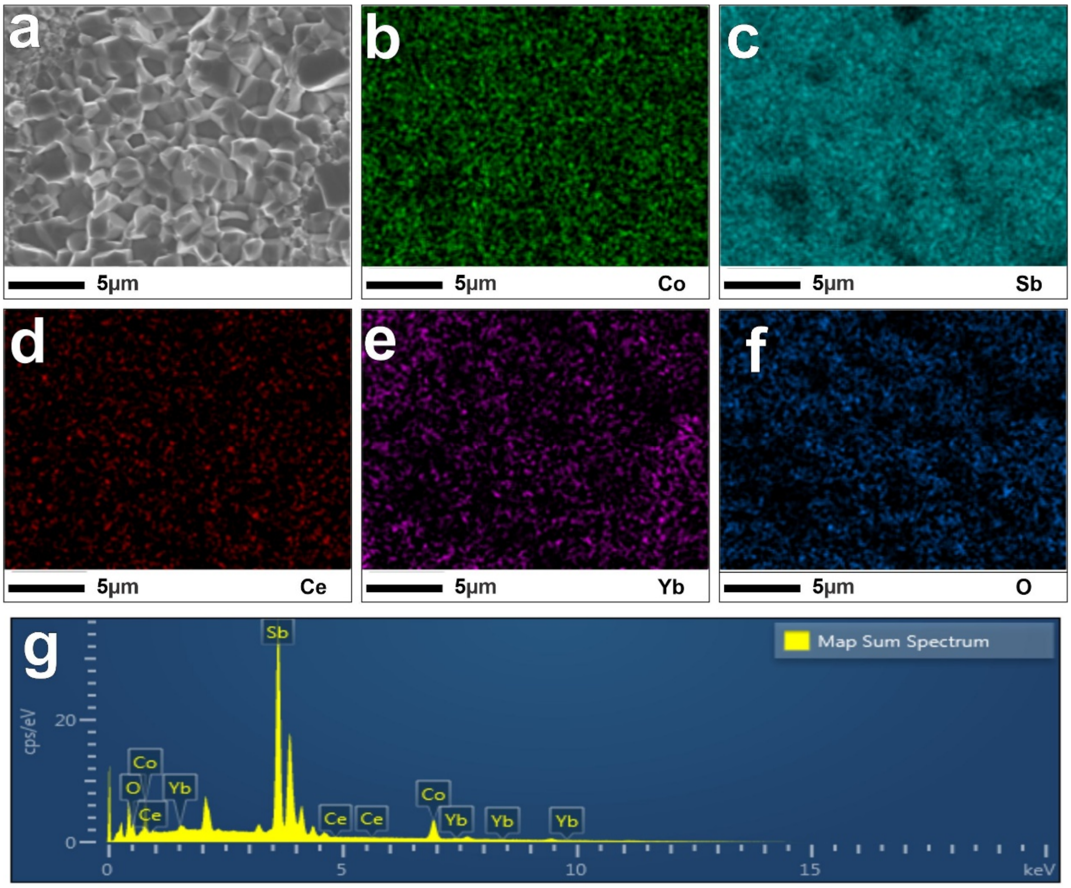

Figure S3: The (a) the SEM image, the distribution of (b) Co, (c) Sb, (d) Ce, (e) Yb, (f) O, and (g) EDS map spectrum data of sample $Ce_{0.05}Yb_{0.15}Co_4Sb_{12}$

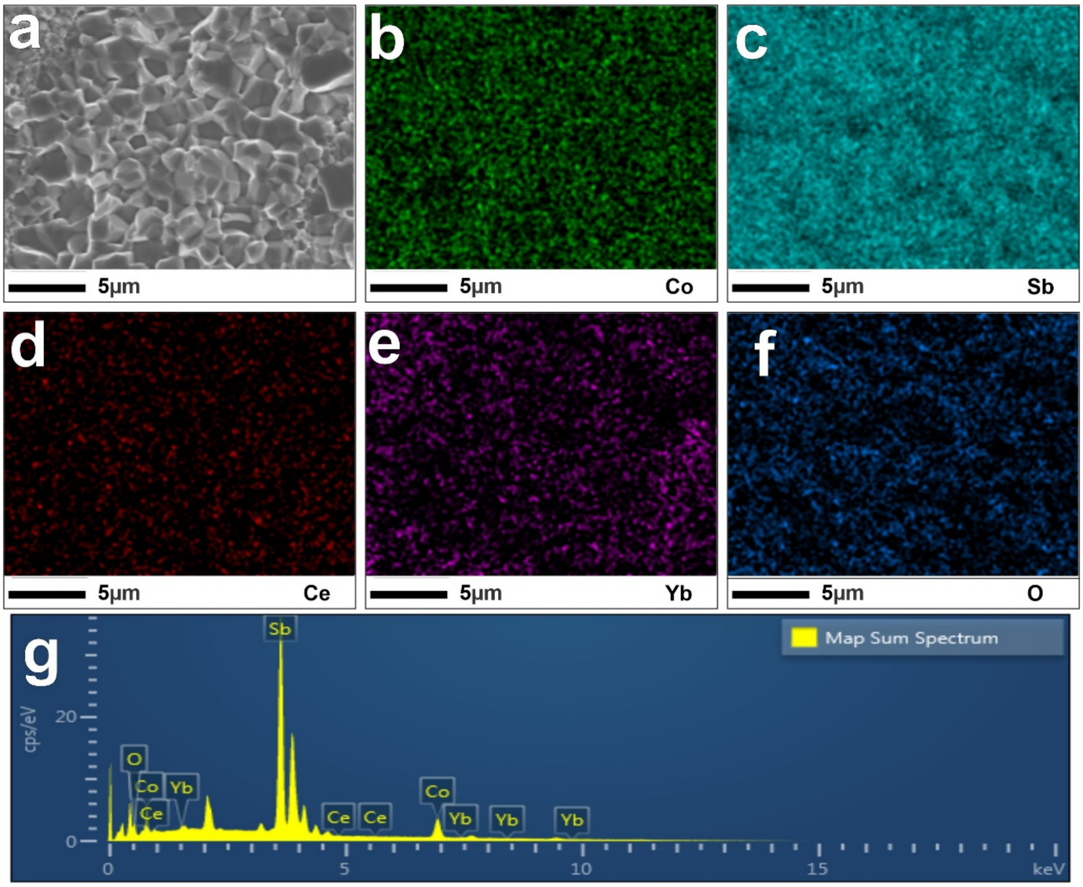

Figure S4: The (a) the SEM image, the distribution of (b) Co, (c) Sb, (d) Ce, (e) Yb, (f) O, and (g) EDS map spectrum data of sample $Ce_{0.075}Yb_{0.125}Co_4Sb_{12}$

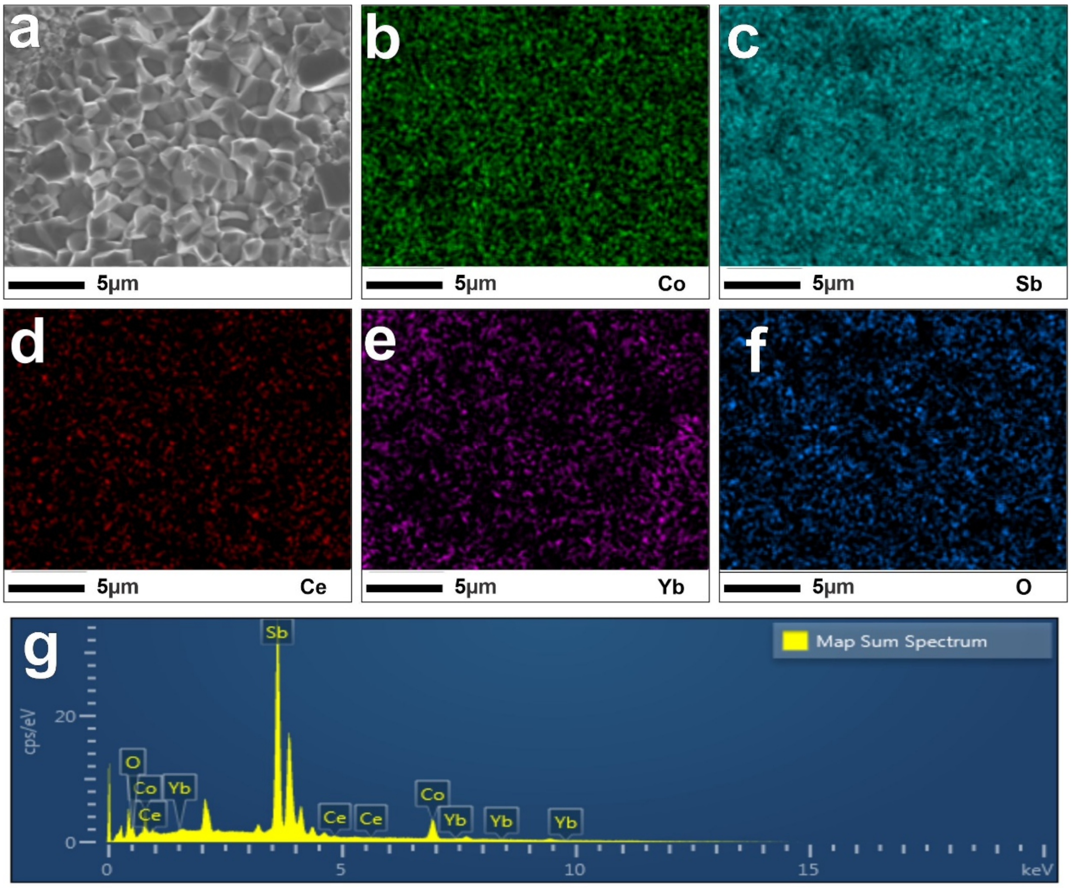

Figure S5: The (a) the SEM image, the distribution of (b) Co, (c) Sb, (d) Ce, (e) Yb, (f) O, and (g) EDS map spectrum data of sample $Ce_{0.1}Yb_{0.1}Co_4Sb_{12}$

Atomic percentage

Ce theoretical	Ce	Yb	Co	Sb	O
0		1.24	21.8	69.32	7.63
0.025	0.03	1.06	22.3	70.21	6.39
0.05	0.26	0.78	22.09	69.75	7.13
0.075	0.32	0.32	22.24	70.59	6.53
0.1	0.74	0.31	22.7	70.2	6.05

Table S2: The atomic percentage of EDS measurement for sample $Ce_xYb_{0.2-x}Co_4Sb_{12}$

Calculated Lorenz number plot

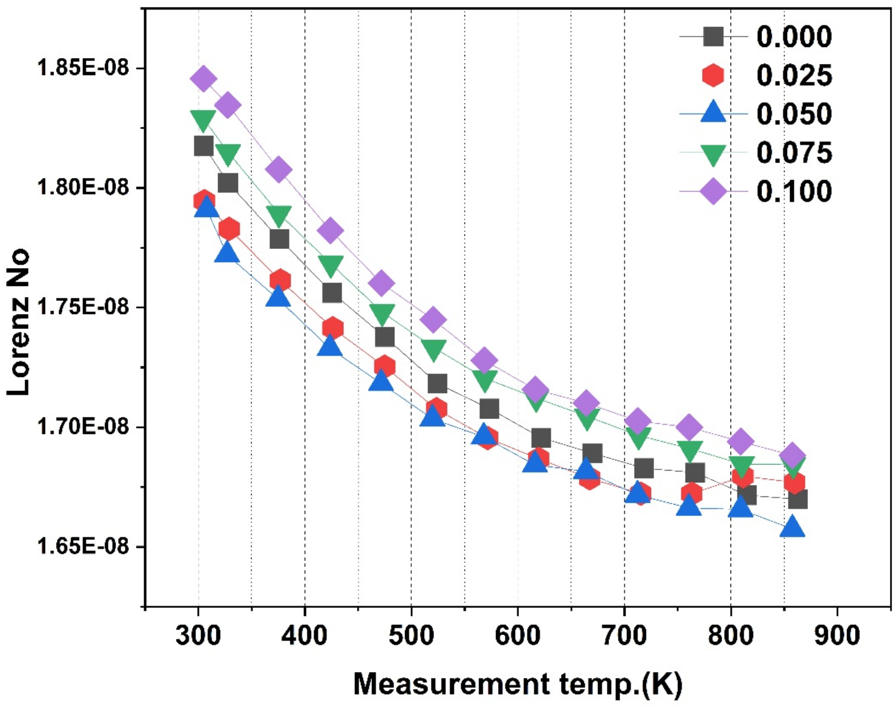

Figure S6: The calculated Lorenz number for sample $Ce_xYb_{0.2-x}Co_4Sb_{12}$
